# Association of cardiovascular health with diabetic complications, all-cause mortality, and life expectancy among people with type 2 diabetes

**DOI:** 10.1186/s13098-022-00934-6

**Published:** 2022-10-28

**Authors:** Yuan Zhang, Rongrong Yang, Yabing Hou, Yanchun Chen, Shu Li, Yaogang Wang, Hongxi Yang

**Affiliations:** 1 School of Public Health, Tianjin Medical University, Tianjin, China; 2 Public Health Science and Engineering College, Tianjin University of Traditional Chinese Medicine, Tianjin, China; 3 Yanjing medical college, Capital Medical University, Beijing, China; 4 School of Public Health, Tianjin Medical University, Tianjin, China; 5 School of Management, Tianjin University of Traditional Chinese Medicine, Tianjin, China; 6 The Discipline of Integrative Medicine, Tianjin University of Traditional Chinese Medicine, Tianjin, China; 7 Department of Bioinformatics, School of Basic Medical Sciences, Tianjin Medical University, Qixiangtai Road 22, Heping District, Tianjin, 300070 China

**Keywords:** Cardiovascular health, Diabetic complications, Mortality, Inflammation marker, Life expectancy, Type 2 diabetes

## Abstract

**Background:**

We aimed to assess the impact of healthy cardiovascular health (CVH) on diabetic complications, mortality, and life expectancy among people with type 2 diabetes and to explore whether inflammation marker mediate these associations.

**Methods:**

This prospective cohort study included 33,236 participants (aged 40–72) with type 2 diabetes from the UK Biobank with annual follow-up from 2006 to 2010 to 2020. Type 2 diabetes was ascertained from self-report, glycated hemoglobin ≥ 6.5%, hospital inpatient registry, or glucose-lowering medication use. Information on mortality was derived from the national death registry. Favorable CVH metrics consisted of non-smoker, regular physical activity, a healthy diet, non-overweight, untreated resting blood pressure < 120/<80 mm Hg, and untreated total cholesterol < 200 mg/dL. Participants were categorized into three groups according to the number of favorable CVH metrics: unfavorable (0 or 1); intermediate (any 2 or 3); and favorable (4 or more). Inflammation marker, as measured by C-reactive protein (CRP), was assessed at baseline and categorized as low (≤ 3 mg/L) and high (> 3 mg/L). Data were analyzed using Cox regression models, flexible parametric survival models, and mediation models.

**Results:**

During the follow-up (median: 11.7 years), 3133 (9.4%) cases of diabetes complications and 4701 (14.1%) deaths occurred. Compared to unfavorable CVH, favorable CVH was associated with a reduced risk of diabetes complications (HR, 0.35; 95% CI, 0.26–0.47) and all-cause mortality (HR, 0.53; 95% CI, 0.43–0.65). In participants with unfavorable CVH, life expectancy at age 45 had a significantly reduction of 7.20 (95% CI, 5.48–8.92) years compared to those with a favorable CVH. Among people with type 2 diabetes, the proportions of diabetes complications and all-cause mortality that would be reduced by promoting the favorable CVH was 61.5% and 39.1%, respectively. CRP level mediated 14.3% and 29.7% of the associations between CVH and diabetic complication and all-cause mortality, respectively.

**Conclusion:**

A favorable CVH was associated with lower risk of diabetes complications and mortality risk, and was associated with a longer life expectancy among people with type 2 diabetes. This association may be in part accounted for by inflammatory processes. Our findings highlight the importance of favorable CVH for the prevention of diabetic complications and all-cause mortality among people with type 2 diabetes, and underscores the need to monitor inflammation among people with unfavorable CVH.

**Supplementary Information:**

The online version contains supplementary material available at 10.1186/s13098-022-00934-6.

## Background

Diabetes is one of the fastest growing diseases worldwide, and is among the top 10 causes of death. Type 2 diabetes is the most common type of diabetes, accounting for around 90% of all diabetes cases. In 2021, the IDF Diabetes Atlas estimated that about 537 million adults are living with diabetes worldwide [1]. Previous studies reported that type 2 diabetes can lead to serious diseases affecting the heart and blood vessels, eyes, kidneys, and nerves, increasing the risk of mortality and decreasing overall quality of life [2–4]. Identifying health behaviors and health factors that lower the risks of diabetic complications and mortality in people with type 2 diabetes is paramount to optimizing health and longevity.

In 2010, the American Heart Association (AHA) developed 7 ideal cardiovascular health (CVH) metrics to stay healthy [5]. international studies have shown a beneficial effect of favorable CVH on cardiovascular disease and mortality [6, 7]. Additionally, Wang et al. suggested that favorable CVH is associated with a lower risk of cardiovascular disease among individuals with prediabetes or diabetes [8]. But it is still unclear whether favorable CVH could mitigate the risk of diabetic complications and mortality among individuals with type 2 diabetes. Besides, Life expectancy is considered to be a common metric for establishing public health priorities, and it is more likely to be understood by wider audiences and convey more intuitive risk messages to the general public [9]. However, evidence on the relationship between CVH and life expectancy among people with type 2 diabetes is limited.

Inflammation has been proposed as an underlying pathophysiological mechanism linking risk factors (e.g., diet pattern, smoking) or metabolic disorders (e.g., dyslipidemia) to an increased risk of non-communicable diseases [10]. Favorable CVH is associated with a lower inflammatory profile [11]. Previous studies have also found that inflammation plays a key role in diabetes complications and mortality [12–14]. However, whether inflammation markers mediated the association between CVH and the risk of diabetes complications and mortality remains unclear. In the present study, among people with type 2 diabetes, we aimed to (1) examine the association between CVH and the risk of diabetic complications, mortality, and life expectancy, and (2) assess the possible mediating role of inflammation in these associations.

## Methods

### Study design and population

The UK Biobank is a population-based cohort study that recruited more than 500,000 participants who attended one of 22 assessment centers across the United Kingdom between 2006 and 2010, and were followed up to 2020 [15]. In this cohort study, among 502,507 participants, a total of 469,271 were excluded, including 462,388 without type 2 diabetes, 698 with prevalent diabetic complications, 6129 with missing data on smoking status, alcohol consumption, physical activity, diet, body mass index (BMI), blood cholesterol, and blood pressure factors at baseline, and 56 lost to follow-up evaluations. Finally, 33,236 participants were available for the current study (Additional file 1: Fig. S1).

### Data collection

Information on sex, age, ethnicity, education, socioeconomic status, employment status, diabetes duration, and family history of diabetes was collected through a touchscreen questionnaire and interview. Ethnicity was categorized as White, Black or Black British, Asian or Asian British, and other. Education was defined as college or university, upper secondary, lower secondary, vocational, and other. Socioeconomic status was defined based on the Townsend deprivation index [16] (encompassing information on social class, employment, car availability, housing) and categorized as low (highest quintile), middle (quintiles 2 to 4), and high (lowest quintile) [17]. Employment status was categorized as working, unemployed, retired, and other. Alcohol consumption was defined based on self-reported intake of red wine, white wine, beer, spirits, and fortified wine and categorized as never/moderate (0 to 14 g/d alcohol for women and 0 to 28 g/d alcohol for men) and non-moderate (> 14 g/d alcohol for women and > 28 g/d alcohol for men) [18]. Information on sugar sweetened beverages was assessed using a touchscreen questionnaire. Participants who reported never consumes drinks containing sugar was defined as not consuming sugar sweetened beverages [19]. Information on triglyceride, serum creatinine, CRP levels, white blood cell (leukocyte) counts, and platelet counts were obtained from blood samples collected at study recruitment. The CRP levels was categorized into two groups (low: ≤3 mg/L; high: >3 mg/L) [20]. Leukocyte counts was coded as low (≤ 10 × 109/L) and high (> 10 × 109/L). Likewise, platelet counts was divided into two groups (low: ≤450 × 109/L; high: >450 × 109/L).

The UK Biobank has full ethical approval from the North West Multi-Center Research Ethics Committee, the National Information Governance Board for Health and Social Care in England and Wales, and the Community Health Index Advisory Group in Scotland. All the UK Biobank participants gave written informed consent before data collection (http://www.ukbiobank.ac.uk/ethics/).

### Diagnosis of type 2 diabetes and its complications

Type 2 diabetes was ascertained based on self-report, medical records, glycated hemoglobin ≥ 6.5%, hospital inpatient registers (International Classification of Diseases [ICD-10] code E11), or use of hypoglycemic agents.

Diabetes complications were assessed using the ICD codes derived from the hospital inpatient registers. The major diabetes complications included renal complications (ICD-10 code E11.2), ophthalmic complications (ICD-10 code E11.3), neurological complications (ICD-10: E11.4), and peripheral circulatory complications (ICD-10 code E11.5).

### Diagnosis of all-cause mortality

Information on all-cause mortality was derived through linkage to national death registries (updated up to November 31, 2020), which included the date and cause of death.

### Assessment of CVH metrics

We defined 6 CVH metrics following the AHA’s recommendations, including smoking status, physical activity, diet, BMI, blood pressure, and cholesterol [5].

Information on smoking status and physical activity was obtained from the Touchscreen questionnaire, diet was derived from the Food Frequency questionnaire, BMI and blood pressure were obtained from physical measurement, and cholesterol concentration was collected from blood sample measured at recruitment. Smoking status was dichotomized as non-smoker vs. being a smoker. Physical activity was measured as the sum of minutes performing walking, moderate and vigorous activity using the International Physical Activity Questionnaire. Regular physical activity was defined as participants who had moderate activity ≥ 150 min per week or vigorous activity ≥ 75 min per week or ≥ 150 min/week of moderate and vigorous activity [5]. A healthy diet score was generated based on the seven commonly eaten food groups (fruits, vegetables, whole grains, refined grains, fish, unprocessed meat, and processed meat) following recommendations on dietary priorities for cardiometabolic health [21]. A healthy diet was based on adequate intake of at least four of seven commonly eaten food groups (1. Fruits: ≥ 3 servings/day; 2. Vegetables: ≥ 3 servings/day; 3. Fish: ≥2 servings/week; 4. Processed meats: ≤ 1 serving/week; 5. Unprocessed red meats: ≤1.5 servings/week; 6. Whole grains: ≥ 3servings/day; 7. Refined grains: ≤1.5 servings/day) [18]. BMI was calculated as weight (kg) divided by height squared (m^2^), and was categorized as non-overweight (< 25 kg/m^2^) and overweight (≥ 25 kg/m^2^). The mean of two measurements was used, and elevated blood pressure was defined as systolic blood pressure (SBP) ≥ 120 mmHg or diastolic blood pressure (DBP) ≥ 80 mmHg, or use of anti-hypertension agents. Ideal cholesterol was defined as untreated total cholesterol < 200 mg/dL. Table 1 in the Additional file 1 provides additional details regarding the assessment of favorable CVH metrics.

**Table 1:** Baseline characteristics of participants by cardiovascular health metrics

Characteristic	Cardiovascular health metrics			*P*–value
	Unfavorable	Intermediate	Favorable	
n (%)	16,585 (48.87)	15,549 (45.82)	1800 (5.30)	
Age, mean (SD), year	59.52 (7.13)	59.3 (7.34)	57.84 (8.04)	< 0.001
Male, n (%)	11,129 (67.10)	8034 (51.67)	468 (26.00)	< 0.001
Education level, n (%)				
College or University	3562 (21.48)	4260 (27.4)	756 (42)	< 0.001
Upper secondary	1497 (9.03)	1542 (9.92)	197 (10.94)	
Lower secondary	4137 (24.94)	3922 (25.22)	419 (23.28)	
Vocational	1482 (8.94)	1252 (8.05)	82 (4.56)	
Other	5907 (35.62)	4573 (29.41)	346 (19.22)	
Socioeconomic status, n (%)^a^				< 0.001
High	2351 (14.18)	2673 (17.19)	413 (22.94)	
Middle	8956 (54)	8969 (57.68)	1083 (60.17)	
Low	5278 (31.82)	3907 (25.13)	304 (16.89)	
Ethnicity, n (%)				< 0.001
White	14,992 (90.39)	13,424 (86.33)	1601 (88.94)	
Asian	806 (4.86)	1027 (6.6)	98 (5.44)	
Black	420 (2.53)	623 (4.01)	37 (2.06)	
Other	367 (2.21)	475 (3.05)	64 (3.56)	
Current employment status, n (%)				0.249
Worked	9392 (56.63)	8889 (57.17)	1021 (56.72)	
Retired	5577 (33.63)	5121 (32.93)	591 (32.83)	
Unemployed	280 (1.69)	274 (1.76)	45 (2.5)	
Other	1336 (8.06)	1265 (8.14)	143 (7.94)	
Alcohol consumption, n (%)				< 0.001
Excessive	8416 (50.81)	7303 (47.03)	788 (43.78)	
Never/moderate	8147 (49.19)	8225 (52.97)	1012 (56.22)	
Smoking status, n (%)				< 0.001
Smoker	11,639 (70.48)	3978 (25.63)	190 (10.56)	
Non–smoker	4874 (29.52)	11,542 (74.37)	1610 (89.44)	
Physical activity, n (%)				
Inactive	11,293 (77.89)	4759 (32.57)	250 (14.2)	< 0.001
Active	3205 (22.11)	9853 (67.43)	1511 (85.8)	
Diet, n (%)				< 0.001
Unhealthy	13,471 (82.22)	5678 (36.74)	262 (14.58)	
Healthy	2914 (17.78)	9778 (63.26)	1535 (85.42)	
BMI (kg/m^2^)				< 0.001
≥ 25 (overweight)	15,742 (97.75)	12,537 (82.12)	363 (20.34)	
< 25 (non–overweight)	362 (2.25)	2729 (17.88)	1422 (79.66)	
Sugar–sweetened beverages, n (%)^b^	906 (5.46)	843 (5.42)	77 (4.28)	< 0.001
Family history of diabetes, n (%)	6906 (41.64)	6537 (42.04)	581 (32.28)	< 0.001
DBP, median (IQR), mm Hg	83 (76–90)	82 (75–89)	76 (69–84)	< 0.001
DBP, median (IQR), mm Hg	143 (132–156)	143 (131–156)	133 (117–151)	< 0.001
Cholesterol, median (IQR), mmol/L	4.6 (3.92–5.54)	4.8 (4.04–5.69)	4.54 (3.65–5.29)	< 0.001
Triglyceride, median (IQR), mmol/L	2.00 (1.49–2.86)	1.75 (1.25–2.49)	1.19 (0.84–1.71)	< 0.001
C–reactive protein, median (IQR), mg/L	2.47 (1.17–4.55)	1.88 (0.88–3.47)	0.90 (0.46–1.89)	< 0.001
Serum creatinine, median (IQR), umol/L	72.3 (63.4–83.8)	71.2 (61.1–80.9)	65.2 (57.6–73.8)	< 0.001

*BMI* body mass index, *DBP* diastolic blood pressure, *IQR* interquartile range, *SBP* systolic blood pressure, *SD* standard deviation

^a^Socioeconomic status was defined based on the Townsend deprivation index (encompassing information on social class, employment, car availability, housing) and categorized as low (highest quintile), middle (quintiles 2 to 4), or high (lowest quintile)

^b^Sugar-sweetened beverages was summarized as the proportion of people who report consuming sugar sweetened beverages

In the current study, favorable CVH metrics included non-smoker, regular physical activity, a healthy diet, non-overweight, untreated resting blood pressure < 120/<80 mm Hg, and untreated total cholesterol < 200 mg/dL. Participants were categorized into three groups according to the number of healthy CVH metrics: (1) unfavorable: participants who had no or only one healthy CVH metric; (2) intermediate: those who had any two healthy CVH metrics; and (3) favorable: those who had 4 or more healthy CVH metrics.

### Statistical analyses

Baseline characteristics of the samples were summarized across CVH categories as percentages for categorical variables and means and standard deviations (SDs) for continuous variables with a normally distribution. If continuous variables did not follow a normal distribution, the variables were summarized as median and interquartile range (IQR). A Shapiro–Wilk normality test was used to assess the normality of the distribution. The ANOVA was used to compare the means of continuous variables and normally distributed data; otherwise, the Mann–Whitney U–test was applied. Categorical data were assessed by chi-square test. Person-years were calculated from the date of recruitment to the date of the death or censoring date, whichever event occurred first. Incidence rates (IRs) per 1000 person-years were calculated for each CVH category.

We used multivariate Cox proportional-hazard models to estimate the hazard ratio (HR) and 95% confidence interval (CI). The proportional hazards assumptions for the Cox model were tested using Schoenfeld residuals method, no violation of the assumption was observed. The duration of follow-up was calculated as a timescale between the baseline assessment and the first event of diabetic complications, death, or last study visit (November 31, 2020). To quantify the contribution of CVH metrics to diabetic complications and all-cause mortality incidence, we calculated the population attributable fraction (PAF), which is the estimated proportional reduction in diabetic complications and all-cause mortality that would occur if CVH metrics were favorable. We estimated the PAF for favorable CVH metrics by the punafcc function in STATA, which was specifically developed for time to event studies.

We further calculated the years of life expectancy lost due to unfavorable CVH. The calculation of years of life lost (i.e. difference in life expectancy) involved a two-step process using flexible parametric survival models with age as the time scale [22]. First, residual life expectancy was estimated as the area under the survival curve up to 100 years old, conditional on surviving at ages 40 to 100 years old (one-year intervals). Second, years of life lost were calculated as the difference between the areas under two survival curves [23–25]. To calculate life expectancy, proportional hazard survival analyses were conducted with the stpm2 command which uses restricted cubic splines to model the baseline cumulative hazard.

 To estimate and quantify the mediation effect of inflammation on the association between CVH at baseline and diabetic complications and all-cause mortality, causal mediation analyses was conducted. Causal mediation analyses using Cox models measure the effect of the exposure on the outcome (total effect, TE) decomposed into natural direct effect (NDE) and natural indirect effect (NIE) [26]. We performed analysis following the previous procedure [27]: (1) simulated multivariable logistic model for the association between CVH and inflammation marker. (2) performed full adjusted Cox models to measure the effect inflammation marker and CVH on the development of diabetic complications and all-cause mortality; (3) calculate the hazard ratios with the coefficient of previous models. Variances and 95% CI calculated using a resampling method that takes random draws from the multivariate normal distribution of estimates [28]. C-reactive protein (CRP) is an exquisitely sensitive systemic marker of inflammation [13, 14]. In the present study, CPR was used as one of the main inflammatory markers to perform mediation analyses. Additionally, considering opportunistic and arbitrary, we also examined other inflammatory markers, such as leukocyte counts and platelet counts to conduct the mediation analysis. The Cox proportional-hazard model, proportional hazard survival analysis, and mediation analysis were adjusted for sex, age, education level, socioeconomic status, ethnic background, alcohol consumption, sugar-sweetened beverages, family history of diabetes, triglyceride, serum creatinine, and CRP. If missing values on covariates, we used multiple imputations based on five replications and a chained-equation method to impute data [29]. Detailed information on missing data was shown in Additional file 1: Table S2.

Several additional analyses were performed to assess the robustness of our study results. First, we used stratification analysis to examine whether the association between CVH and diabetic complications and all-cause mortality varied by sex, age, socioeconomic status, alcohol consumption, or family history of diabetes. Next, we constructed a weighted CVH score based on the six favorable CVH metrics using the following equation: weighted CVH score = (β_1_*factor 1 + β_2_*factor 2 + β_3_*factor 3 + β_4_* factor 4 + β_5_*factor 5 + β_6_*factor 6) (6/sum of the coefficients). This weighted score ranged from 0 to 6 points and factors in the magnitudes of the adjusted relative risk for each factor. Additionally, to address the role of potential reverse causality, we repeated the main analyses in a sample excluding participants who developed incident diabetic complications or deaths within the first 3-year follow-up period. We repeated the main analyses with additional adjustment for diabetes duration. Finally, we performed the analysis in overall participants (including participants with and without diabetes) to examined the association between CVH and the risk of mortality and life expectancy.

All the analyses were performed using STATA 15 statistical software (Stata Corp, College Station, TX, USA) and R (version 3.6.1, R Foundation for Statistical Computing). All *P*-values were two-sided, with statistical significance set at 0.05.

## Results

Of the 33,236 study participants, 19,128 (57.6%) were men, and the mean (SD) age was 59.3 (7.3) years. Over a median of 11.7 years (377,250 person-years) of follow-up, 382 participants (1.2%) developed renal complications, 2239 participants (6.7%) developed ophthalmic complications, 830 participants (2.5%) developed neurological complications, 464 participants (1.4%) developed peripheral circulatory complications, and 4701 (14.2%) participants recorded deaths. Compared to participants with an unfavorable CVH, those with an unfavorable CVH were more likely to be older, whites, smoker, physical inactive, non-moderate alcohol consumption; and to have higher DBP, SBP, cholesterol, triglyceride, CRP, and serum creatinine levels and lower educational attainment and socioeconomic status (Table 1).

In multi-adjusted Cox regression models, compared to an unfavorable CVH, a favorable CVH was associated with a reduced risk of diabetic complications (HR, 0.35; 95% CI, 0.26–0.47) (Table 2). In addition, the HR (95% CI) for diabetic complications per 1 point increment in favorable CVH metrics was 0.87 (95% CI, 0.84–0.90), for renal complications was 0.83 (95% CI, 0.75–0.92), for ophthalmic complications was 0.89 (95% CI, 0.85–0.93), for neurological complications was 0.83 (95% CI, 0.77–0.87), and for peripheral circulatory complications was 0.81 (95% CI, 0.73–0.89). Additionally, the proportions of diabetes complications that would be reduced by promoting the favorable CVH was 61.5%.

**Table 2:** Hazard ratios (HRs) and 95% confidence interval (CI) of diabetes complications by cardiovascular health (CVH) among people with type 2 diabetes

Diabetes complications	No. of event	IR^a^	Basic–adjusted HR (95% CI)^b^	Multi–adjusted HR (95% CI)^c^
Unfavorable CVH	1785	9.80	1.00 (Ref.)	1.00 (Ref.)
Intermediate CVH	1284	7.37	0.77 (0.72–0.83)	0.85 (0.79–0.91)
Favorable CVH	44	2.11	0.24 (0.18–0.32)	0.35 (0.26–0.47)

^a^Incidence rates are provided per 1000 person-years. ^b^Adjusted for sex and age

^c^Adjusted for sex, age, education level, socioeconomic status, ethnicity background, alcohol consumption, sugar-sweetened beverages, family history of diabetes, triglyceride, serum creatinine, and C-reactive protein

We estimated the association between all-cause mortality and CVH metrics among people with type 2 diabetes (Table 3). In multi-adjusted Cox regression models, compared to an unfavorable CVH, the HRs (95% Cis) of all-cause mortality were 0.73 (0.69–0.78) for an intermediate CVH, and 0.53 (0.43–0.65) for a favorable CVH. The risks of all-cause mortality decreased significantly with the increase of each 1 point increment in favorable cardiovascular health metrics (HR, 0.81, 95% CI, 0.79–0.84). And 39.1% of all-cause mortality could be prevented if all individuals in a population had a favorable CVH.

**Table 3:** Hazard ratios (HRs) and 95% confidence interval (CI) of all-cause mortality by weight cardiovascular health (CVH) among people with type 2 diabetes

All–cause mortality	No. of event	IR^a^	Basic–adjusted HR (95% CI)^b^	Multi–adjusted HR (95% CI)^c^
Unfavorable CVH	2890	16.26	1.00 (Ref.)	1.00 (Ref.)
Intermediate CVH	1707	9.89	0.64 (0.61–0.68)	0.72 (0.68–0.77)
Favorable CVH	104	5.06	0.39 (0.32–0.48)	0.53 (0.43–0.64)

^a^Incidence rates are provided per 1000 person-years.^b^Adjusted for sex and age

^c^Adjusted for sex, age, education level, socioeconomic status, ethnicity background, alcohol consumption, sugar-sweetened beverages, family history of diabetes, diabetes complications, triglyceride, serum creatinine, and C-reactive protein

**Fig. 1:**
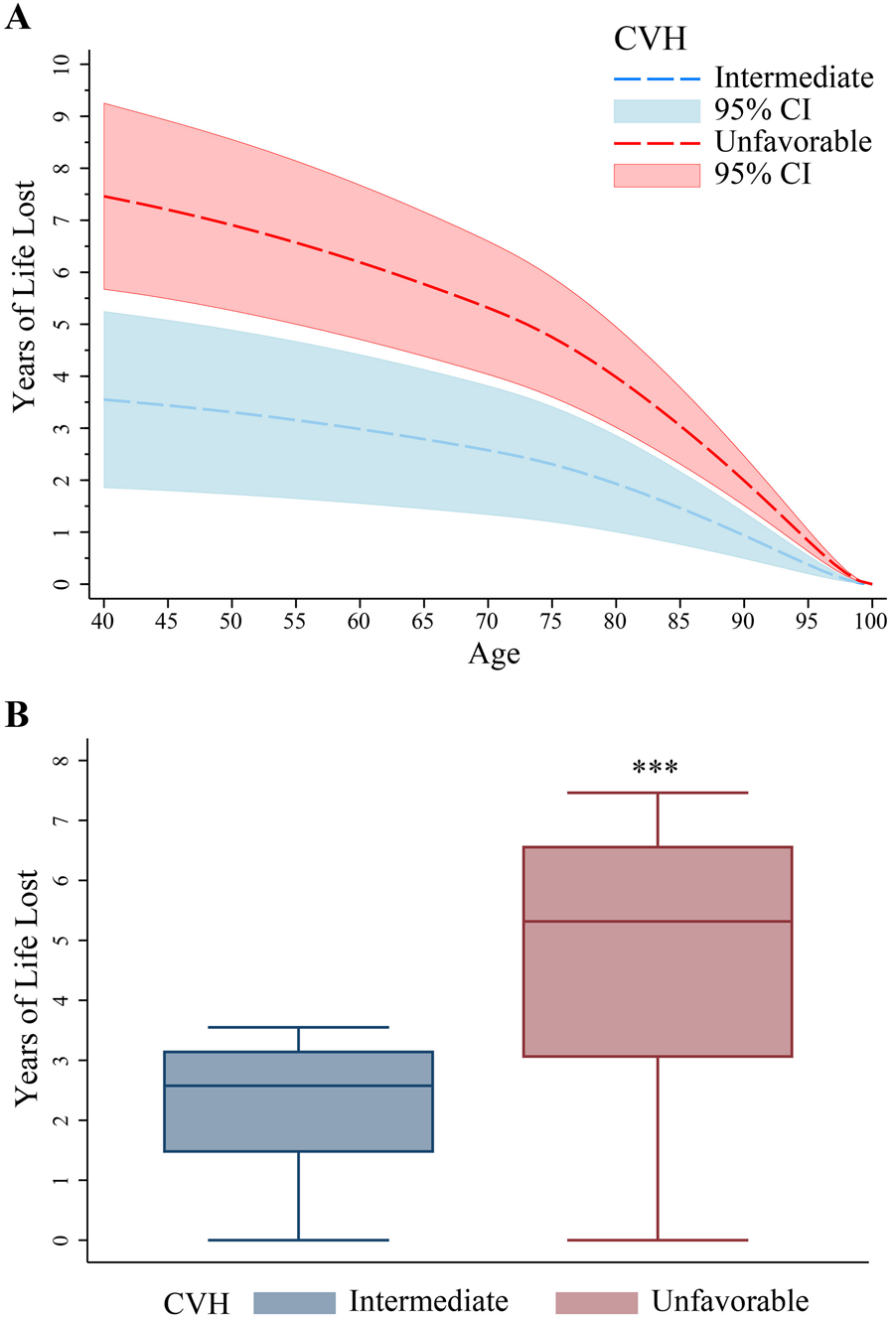
Years of life expectancy lost by cardiovascular health (CVH) metrics among people with type 2 diabetes. **A** Years of life lost were estimated as the difference in residual life expectancy between participants with intermediate CVH and participants with unfavorable CVH; **B** Box plots of the distributions of years of life expectancy lost by intermediate and unfavorable CVH. Model was adjusted for sex, age, education level, socioeconomic status, ethnicity background, alcohol consumption, sugar-sweetened beverages, family history of diabetes, triglyceride, serum creatinine, and C-reactive protein

We also estimated the years of life expectancy loss associated with an intermediate/unfavorable CVH compared to a favorable CVH among participants with type 2 diabetes (Fig. 1a). Comparted to participants with a favorable CVH, life expectancy of those with an intermediate/unfavorable CVH at age 45 had a significantly reduction of 3.44 (95% CI, 1.79–5.08)/7.20 (95% CI, 5.48–8.92) years, and at age 65 had a significantly reduction of 2.79 (95% CI, 1.45–4.13)/5.77 (95% CI, 4.38–7.16) years. The median years of life expectancy loss in the unfavorable CVH group were higher than that in the intermediate CVH group (5.32 years versus 2.58 years, *P* < 0.001) (Fig. 1b).

**Fig. 2:**
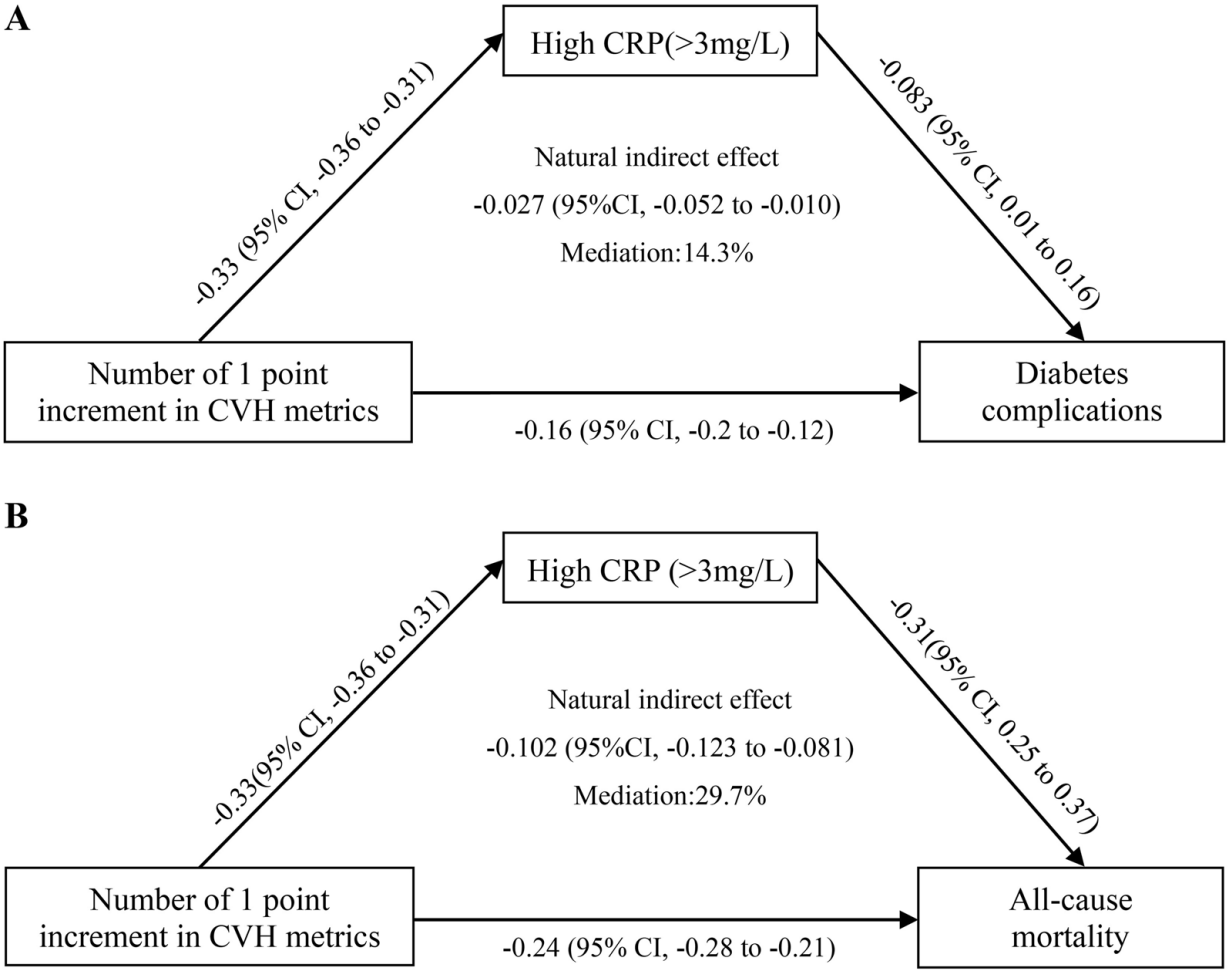
Mediation analysis of inflammation marker, as measured by C-reactive protein level, on the associations of cardiovascular health (CVH) metrics with diabetic complications **A** and all-cause mortality (**B**). The mediation analyses were adjusted for sex, age, education level, socioeconomic status, ethnicity background, alcohol consumption, sugar-sweetened beverages, family history of diabetes, triglyceride, serum creatinine. ^*^P < 0.05

During causal mediation analysis, binary variables were created for CRP (≤ 3.0 mg/L; >3.0 mg/L). The number of 1 point increment in favorable CVH metrics was related to lower CRP (β coefficient=-0.33, 95% CI: − 0.36 to − 0.31). A significant NIE was observed between the number of 1 point increment in favorable CVH metrics and diabetic complications (β coefficient = − 0.027; 95% CI, − 0.052, − 0.010) and all-cause mortality (β =− 0.102; 95% CI, − 0.123, − 0.081), indicating that 14.3% and 29.7% of the associations between CVH and diabetic complication and all-cause mortality were mediated by CRP, respectively (Fig. 2). Similar, leukocyte counts mediated 34.4% (indirect effect: β coefficient = − 0.0084; 95% CI, − 0.119, − 0.051) and 32.2% (indirect effect: β =− 0.119; 95% CI, − 0.149, − 0.091) of the associations between CVH and diabetic complication and all-cause mortality, respectively. Platelet counts mediated 15.3% (indirect effect: β coefficient = − 0.030; 95% CI, − 0.130, − 0.012) and 35.5% (indirect effect: β coefficient = − 0.144; 95% CI, − 0.265, − 0.049) of the associations between CVH and diabetic complication and all-cause mortality, respectively.

Consistent results were observed when we performed stratified analysis by sex, age, socioeconomic status, and alcohol consumption (for diabetic complications and all-cause mortality events; Additional file 1: Fig. S2). We observed an interaction of each 1 point increment in favorable CVH metrics with a family history of diabetes on all-cause mortality. The HR for all-cause mortality was 0.86 (95% CI, 0.81–0.9) among participants who had a family history of diabetes and 0.78 (95% CI, 0.75–0.81) among those without a family history of diabetes (*P* for interaction = 0.023). There were no other significant interactions. In addition, similar results were obtained when we applied a weighted score for CVH metrics (Additional file 1: Tables S2, S3; when we excluded participants who developed incident diabetic complications or those who died within the first 3-year follow-up period (Additional file 1: Tables S4, S5). The results were not much altered compared with those from initial analyses when we repeated analyses with additional adjustment for diabetes duration (Additional file 1: Tables S6, S7 and Fig. S3) or when we performed the analysis for including participants without diabetes (Additional file 1: Tables S8 and Fig. S4).

## Discussion

In this large population-based prospective cohort study, we found that: (1) a favorable CVH could counteract the risk of diabetes complications and mortality among individuals with type 2 diabetes; (2) an unfavorable CVH reduces life expectancy by almost 7 years at age 45 and 6 years at age 65 compared to a favorable CVH; (3) CRP level partly mediated the associations between CVH metrics and diabetic complications and all-cause mortality.

Complications of diabetes, including cardiovascular disease, and microvascular complications, such as diabetic kidney disease, diabetic retinopathy, and neuropathy, are responsible for much of the burden associated with diabetes, with profound effects on quality of life, demand on health services and economic costs. Thus, beyond the glycemic control index, there needs another comprehensive indicator to evaluate the health status of individuals with type 2 diabetes. The China Cardiometabolic Disease and Cancer Cohort Study confirmed that participants with prediabetes or diabetes who had 5 or more favorable CVH metrics exhibited lower or no significant excess cardiovascular disease (CVD) risks compared with the participants with normal glucose regulation [8]. Matthew reported that favorable CVH in childhood and improvement in CVH from childhood to adulthood appears to have a protective effect on the retinal microvasculature in those with, without, and at risk of diabetes mellitus [30]. Our findings showed that a favorable CVH was associated with lower risk of diabetes complications. The CVH metrics provided a positive frame of reference for behaviors and health makers, and are also of value in identifying high-risk individuals and facilitating health management.

Our results confirmed that an unfavorable CVH can increase the risk of mortality and reduce life expectancy in people with type 2 diabetes. Bamba et al. conducted a prospective study, including 9,294 men and women, reported that individuals with 5 or more favorable CVH metrics had a 29% decreased risk of all-cause mortality compared with those with less than 3 favorable CVH metrics [31]. A prospective population-based Kuopio Ischemic Heart Disease cohort study found that men with an favorable CVH score had a 67% lower risk of all-cause mortality compared with men with a poor CVH score [32]. Life expectancy is considered to be a common metric for establishing public health priorities. However, there are few studies concerning the relationship between CVH metrics and life expectancy in individuals with type 2 diabetes. Our study found that an unfavorable CVH reduces life expectancy by almost 7 years at age 45 and 6 years at age 65 compared to a favorable CVH. These findings were more likely to be understood by wider audiences and convey more intuitive risk messages to the general public. The quantitative assessment of life lost due to unhealthy CVH were more likely to be understood by wider audiences and convey more intuitive risk messages to the general public. Our study further highlights the benefits of maintaining better CVH across the life course and calls attention to the need for comprehensive strategies (healthy behavioral lifestyle and biological phenotype) to preserve and restore a higher CVH level. To our knowledge, this is the first prospective study to assess the association of CVH metrics and life expectancy in people with type 2 diabetes.

Available evidence has reported that inflammation is partially overlapping pathophysiological features of unhealthy CVH metrics, diabetic complications, and mortality [10, 13, 14], but no studies have estimated the extent to which inflammatory factor might mediate the associations of CVH metrics with diabetic complications and all-cause mortality. In the current study, among participants with type 2 diabetes, we found that unfavorable CVH was related to a higher CRP level, which in turn was related to a higher development of diabetes complications and mortality.

Our results indicate that CRP explained only a small proportion of the associations, suggesting that several other contributors might lie on the pathway between CVH metrics and diabetic complications and mortality among participants with type 2 diabetes. However, the mechanisms responsible for unfavorable CVH attribute to diabetic complications and mortality are incompletely understood. Endothelial dysfunction may explain the higher incidence of diabetic complications and mortality in people with unfavorable CVH [33–36]. CVH metrics such as BMI, blood pressure, and smoking might affect endothelial function, and conversely, endothelial dysfunction that corresponds with an elevated risk of incident cardiovascular/microvascular disease [37–39]. Thus, prevalent diabetic complications may be a marker of accelerated endothelial dysfunction and indirectly related to the serious health problem prognosis and mortality. Unfavorable CVH was associated with oxidative stress, which plays an indispensable role in the development of cardiovascular disease [40]. In addition, people with type 2 diabetes who adherence to a favorable CVH can reduce insulin resistance and improve glycemic control, thereby reducing the harmful effects of hyperglycemia [41].

This study has several strengths, including a large study sample with a long follow-up period, the use of standardized protocols for data collection, and comprehensive diagnoses using multiple resources (e.g., physical examination, hospital inpatient records, and death registers). Despite these strengths, several limitations of the study need to be considered. Potential changes in CVH factors after the baseline examination may have influenced our risk estimates. Future research is needed to investigate the effects of changes in CVH factors overtime on the observed associations. In addition, although all analyses in the present study were adjusted for known potential sources of bias, the possibility of unmeasured confounding factors and reverse causation remains. Finally, participants in the present study were primarily of white British descent, limiting the generalizability of the findings to Afro-British, Asian British, and other ethnic groups that may be at higher risk for diabetic complications and all-cause mortality.

## Conclusion

In this large population-based cohort study, we found that favorable CVH was associated with lower risks of diabetes complications and all-cause mortality, and was associated with a longer life expectancy among individuals with type 2 diabetes. CRP level may partly account for the associations between CVH metrics and diabetic complications and all-cause mortality. Our findings highlight the importance of favorable CVH for the prevention of diabetic complications and all-cause mortality among people with type 2 diabetes, and underscores the need to monitor inflammation among people with unfavorable CVH.

## Supplementary Information

**Additional file 1:** **Table S1**. Definition of cardiovascular health (CVH) metrics. **Table S2**. Detailed information on missing covariates. **Table S3**. Hazard ratios (HRs) and 95% confidence interval (CI) of diabetic complications by weight cardiovascular health (CVH) among people with type 2 diabetes. **Table S4**. Hazard ratios (HRs) and 95% confidence interval (CI) of all-cause mortality by weight cardiovascular health (CVH) among people with type 2 diabetes. **Table S5**. Hazard ratios (HRs) and 95% confidence interval (CI) of diabetic complications by cardiovascular health (CVH) among people with type 2 diabetes after excluding first 3 years incidence of diabetic complications during follow-up. **Table S6**. Hazard ratios (HRs) and 95% confidence interval (CI) of mortality by cardiovascular health (CVH) among people with type 2 diabetes after excluding those who died within the first 3-year follow-up period. **Table S7**. Hazard ratios (HRs) and 95% confidence interval (CI) of diabetes complications by cardiovascular health (CVH) among people with type 2 diabetes after further adjustment for diabetes duration. **Table S8**. Hazard ratios (HRs) and 95% confidence interval (CI) of all-cause mortality by weight cardiovascular health (CVH) among people with type 2 diabetes after further adjustment for diabetes duration. **Figure S1**. Flowchart for the selection of the analyzed study sample from the UK Biobank study. **Figure S2**. Hazard ratios (HRs) of diabetic complications and all-cause mortality per 1-number increment in ideal cardiovascular health (CVH) metrics according to stratification categories among participants with type 2 diabetes. **Figure S3**. Years of life expectancy lost by cardiovascular health (CVH) metrics among people with type 2 diabetes after further adjustment for diabetes duration. **Figure S4**. Years of life expectancy lost by cardiovascular health (CVH) metrics among people with and without type 2 diabetes.

## Data Availability

The data are available on application to the UK Biobank (www.ukbiobank.ac.uk/).
