# Supplementary material for: Association of cardiovascular health with diabetic complications, all-cause mortality, and life expectancy among people with type 2 diabetes
Source: Diabetol Metab Syndr. 2022 Oct 28;14:158. doi: 10.1186/s13098-022-00934-6 (PMC9615235; doi:10.1186/s13098-022-00934-6)
Supplement: Supplementary file 1 — Additional file 1: Table S1. Definition of cardiovascular health (CVH) metrics. Table S2. Detailed information on missing covariates. Table S3. Hazard ratios (HRs) and 95% confidence interval (CI) of diabetic complications by weight cardiovascular health (CVH) among people with type 2 diabetes. Table S4. Hazard ratios (HRs) and 95% confidence interval (CI) of all-cause mortality by weight cardiovascular health (CVH) among people with type 2 diabetes. Table S5. Hazard ratios (HRs) and 95% confidence interval (CI) of diabetic complications by cardiovascular health (CVH) among people with type 2 diabetes after excluding first 3 years incidence of diabetic complications during follow-up. Table S6. Hazard ratios (HRs) and 95% confidence interval (CI) of mortality by cardiovascular health (CVH) among people with type 2 diabetes after excluding those who died within the first 3-year follow-up period. Table S7. Hazard ratios (HRs) and 95% confidence interval (CI) of diabetes complications by cardiovascular health (CVH) among people with type 2 diabetes after further adjustment for diabetes duration. Table S8. Hazard ratios (HRs) and 95% confidence interval (CI) of all-cause mortality by weight cardiovascular health (CVH) among people with type 2 diabetes after further adjustment for diabetes duration. Figure S1. Flowchart for the selection of the analyzed study sample from the UK Biobank study. Figure S2. Hazard ratios (HRs) of diabetic complications and all-cause mortality per 1-number increment in ideal cardiovascular health (CVH) metrics according to stratification categories among participants with type 2 diabetes. Figure S3. Years of life expectancy lost by cardiovascular health (CVH) metrics among people with type 2 diabetes after further adjustment for diabetes duration. Figure S4. Years of life expectancy lost by cardiovascular health (CVH) metrics among people with and without type 2 diabetes. [file 13098_2022_934_MOESM1_ESM.doc]

**Additional file 1**

**Table S1.** Definition of cardiovascular health (CVH) metrics

**Table S2.** Detailed information on missing covariates

**Table S3.** Hazard ratios (HRs) and 95% confidence interval (CI) of diabetic complications by weight cardiovascular health (CVH) among people with type 2 diabetes

**Table S4.** Hazard ratios (HRs) and 95% confidence interval (CI) of all-cause mortality by weight cardiovascular health (CVH) among people with type 2 diabetes

**Table S5.** Hazard ratios (HRs) and 95% confidence interval (CI) of diabetic complications by cardiovascular health (CVH) among people with type 2 diabetes after excluding first 3 years incidence of diabetic complications during follow-up

**Table S6.** Hazard ratios (HRs) and 95% confidence interval (CI) of mortality by cardiovascular health (CVH) among people with type 2 diabetes after excluding those who died within the first 3-year follow-up period

**Table S7.** Hazard ratios (HRs) and 95% confidence interval (CI) of diabetes complications by cardiovascular health (CVH) among people with type 2 diabetes after further adjustment for diabetes duration

**Table S8.** Hazard ratios (HRs) and 95% confidence interval (CI) of all-cause mortality by weight cardiovascular health (CVH) among people with type 2 diabetes after further adjustment for diabetes duration

**Figure S1.** Flowchart for the selection of the analyzed study sample from the UK Biobank study

**Figure S2.** Hazard ratios (HRs) of diabetic complications and all-cause mortality per 1-number increment in ideal cardiovascular health (CVH) metrics according to stratification categories among participants with type 2 diabetes

**Figure S3.** Years of life expectancy lost by cardiovascular health (CVH) metrics among people with type 2 diabetes after further adjustment for diabetes duration

**Figure S4.** Years of life expectancy lost by cardiovascular health (CVH) metrics among people with and without type 2 diabetes

**Table S1. Definition of cardiovascular health (CVH) m**etrics

| Metrics | Unfavorable level | Favorable level |
| --- | --- | --- |
| Smoking status | Current or former smoker | Never smoked |
| Body mass index | ≥ 25 kg/m^2^ | BMI < 25 kg/m^2^ |
| Diet pattern | 0-3 of the following 7 food groups:  1. Fruits: ≥ 3 servings/day  2. Vegetables: ≥ 3 servings/day  3. Fish: ≥2 servings/week  4. Processed meats: ≤ 1 serving/week  5.Unprocessed red meats: ≤1.5 servings/week  6. Whole grains: ≥ 3 servings/day  7. Refined grains: ≤1.5 servings/day | At least 4 of the following 7 food groups:  1. Fruits: ≥ 3 servings/day  2. Vegetables: ≥ 3 servings/day  3. Fish: ≥2 servings/week  4. Processed meats: ≤ 1 serving/week  5.Unprocessed red meats: ≤1.5 servings/week  6. Whole grains: ≥ 3 servings/day  7. Refined grains: ≤1.5 servings/day |
| Physical activity | 0-149 min/week of moderate activity OR 0-74 min/week of vigorous activity OR 0-149 min/week of moderate and vigorous activity | ≥150 min/week of moderate activity OR ≥75 min/week of vigorous activity OR ≥150 min/week of moderate and vigorous activity |
| Serum cholesterol | ≥200 mg/dL OR treated | <200 mg/dL |
| Blood pressure | SBP ≥120 mm Hg OR DBP ≥80 mm Hg OR treated | SBP <120 mm Hg and DBP <80 mm Hg |

DBP =diastolic blood pressure; SBP = systolic blood pressure.

**Table S2. Detailed information on** missing covariates

| Covariates | No. of participants | Missing rate (%) |
| --- | --- | --- |
| Education level | 32634 | 1.82 |
| Socioeconomic status | 33185 | 0.15 |
| Ethnicity background | 33072 | 0.50 |
| Alcohol consumption | 33185 | 0.15 |
| Triglyceride | 31725 | 4.76 |
| Serum creatinine | 31754 | 4.67 |
| C-reactive protein | 31678 | 4.92 |

**Table S3. Hazard ratios (HRs) and 95% confidence interval (CI) of diabetic complications by weight cardiovascular health (CVH) among people with type 2 diabetes**

| **Diabetes complications** | No. of  event | IR^a^ | Basic-adjusted  HR (95% CI)^b^ | Multi-adjusted  HR (95% CI)^c^ |
| --- | --- | --- | --- | --- |
| Unfavorable CVH | 1791 | 9.77 | 1.00 (Ref.) | 1.00 (Ref.) |
| Intermediate CVH | 972 | 7.38 | 0.78 (0.72-0.85) | 0.85 (0.79-0.92) |
| Favorable CVH | 350 | 5.62 | 0.60 (0.54-0.68) | 0.72 (0.64-0.81) |
| Per 1-number increment in ideal CVH factors | 3113 | 8.25 | 0.87 (0.84-0.89) | 0.91 (0.88-0.94) |

^a^Incidence rates are provided per 1000 person-years.

^b^Adjusted for sex and age.

^c^Adjusted for sex, age, education level, socioeconomic status, ethnicity background, alcohol consumption, sugar-sweetened beverages, family history of diabetes, diabetes complications, triglyceride, serum creatinine, and C-reactive protein.

**Table S4. Hazard ratios (HRs) and 95% confidence interval (CI) of all-cause mortality by weight cardiovascular health (CVH) among people with type 2 diabetes**

| **All-cause mortality** | No. of  event | IR^a^ | Basic-adjusted  HR (95% CI)^b^ | Multi-adjusted  HR (95% CI)^c^ |
| --- | --- | --- | --- | --- |
| Unfavorable CVH | 3077 | 16.71 | 1.00 (Ref.) | 1.00 (Ref.) |
| Intermediate CVH | 1723 | 10.17 | 0.65 (0.61-0.69) | 0.73 (0.68-0.77) |
| Favorable CVH | 141 | 5.98 | 0.44 (0.37-0.52) | 0.56 (0.47-0.67) |
| Per 1-number increment in ideal CVH factors | 4941 | 13.10 | 0.80 (0.78-0.82) | 0.86 (0.83-0.88) |

^a^Incidence rates are provided per 1000 person-years.

^b^Adjusted for sex and age.

^c^Adjusted for sex, age, education level, socioeconomic status, ethnicity background, alcohol consumption, sugar-sweetened beverages, family history of diabetes, diabetes complications, triglyceride, serum creatinine, and C-reactive protein.

**Table S5. Hazard ratios (HRs) and 95% confidence interval (CI) of diabetic complications by cardiovascular health (CVH) among people with type 2 diabetes after excluding first 3 years incidence of diabetic complications** during follow-up

| **Diabetes complications** | No. of  event | IR^a^ | Basic-adjusted  HR (95% CI)^b^ | Multi-adjusted  HR (95% CI)^c^ |
| --- | --- | --- | --- | --- |
| Unfavorable CVH | 1440 | 8.13 | 1.00 (Ref.) | 1.00 (Ref.) |
| Intermediate CVH | 1071 | 6.25 | 0.79 (0.73-0.85) | 0.86 (0.79-0.93) |
| Favorable CVH | 36 | 1.74 | 0.23 (0.17-0.33) | 0.35 (0.25-0.49) |
| Per 1-number increment in ideal CVH factors | 2547 | 6.90 | 0.81 (0.78-0.84) | 0.87 (0.84-0.91) |

^a^Incidence rates are provided per 1000 person-years.

^b^Adjusted for sex and age.

^c^Adjusted for sex, age, education level, socioeconomic status, ethnicity background, alcohol consumption, sugar-sweetened beverages, family history of diabetes, diabetes complications, triglyceride, serum creatinine, and C-reactive protein.

**Table S6. Hazard ratios (HRs) and 95% confidence interval (CI) of mortality by cardiovascular health (CVH) among people with type 2 diabetes after excluding those who died within the first 3-year follow-up period**

| **Diabetes complications** | No. of  event | IR^a^ | Basic-adjusted  HR (95% CI)^b^ | Multi-adjusted  HR (95% CI)^c^ |
| --- | --- | --- | --- | --- |
| Unfavorable CVH | 2483 | 15.76 | 1.00 (Ref.) | 1.00 (Ref.) |
| Intermediate CVH | 1487 | 9.87 | 0.64 (0.60-0.69) | 0.71 (0.66-0.76) |
| Favorable CVH | 93 | 5.23 | 0.40 (0.32-0.49) | 0.49 (0.40-0.61) |
| Per 1-point increment in ideal CVH factors | 4063 | 6.90 | 0.75 (0.73-0.78) | 0.80 (0.77-0.83) |

^a^Incidence rates are provided per 1000 person-years.

^b^Adjusted for sex and age.

^c^Adjusted for sex, age, education level, socioeconomic status, ethnicity background, alcohol consumption, sugar-sweetened beverages, family history of diabetes, diabetes complications, triglyceride, serum creatinine, and C-reactive protein.

**Table S7. Hazard ratios (HRs) and 95% confidence interval (CI) of diabetes complications by cardiovascular health (CVH) among people with type 2 diabetes after further adjustment for diabetes duration**

| Diabetes complications | No. of  event | IR^a^ | Basic-adjusted  HR (95% CI)^b^ | Multi-adjusted  HR (95% CI)^c^ |
| --- | --- | --- | --- | --- |
| Unfavorable CVH | 1785 | 9.80 | 1.00 (Ref.) | 1.00 (Ref.) |
| Intermediate CVH | 1284 | 7.37 | 0.77 (0.72-0.83) | 0.82 (0.77-0.89) |
| Favorable CVH | 44 | 2.11 | 0.24 (0.18-0.32) | 0.32 (0.24-0.44) |

^a^Incidence rates are provided per 1000 person-years.

^b^Adjusted for sex and age.

^c^Adjusted for sex, age, education level, socioeconomic status, ethnicity background, alcohol consumption, sugar-sweetened beverages, family history of diabetes, triglyceride, serum creatinine, C-reactive protein, and diabetes duration.

**Table S8. Hazard ratios (HRs) and 95% confidence interval (CI) of all-cause mortality by weight cardiovascular health (CVH) among people with type 2 diabetes** after further adjustment for diabetes duration

| All-cause mortality | No. of  event | IR^a^ | Basic-adjusted  HR (95% CI)^b^ | Multi-adjusted  HR (95% CI)^c^ |
| --- | --- | --- | --- | --- |
| Unfavorable CVH | 2890 | 16.26 | 1.00 (Ref.) | 1.00 (Ref.) |
| Intermediate CVH | 1707 | 9.89 | 0.64 (0.61-0.68) | 0.71 (0.67-0.75) |
| Favorable CVH | 104 | 5.06 | 0.39 (0.32-0.48) | 0.49 (0.40-0.60) |

^a^Incidence rates are provided per 1000 person-years.

^b^Adjusted for sex and age.

^c^Adjusted for sex, age, education level, socioeconomic status, ethnicity background, alcohol consumption, sugar-sweetened beverages, family history of diabetes, diabetes complications, triglyceride, serum creatinine, C-reactive protein, and diabetes duration.


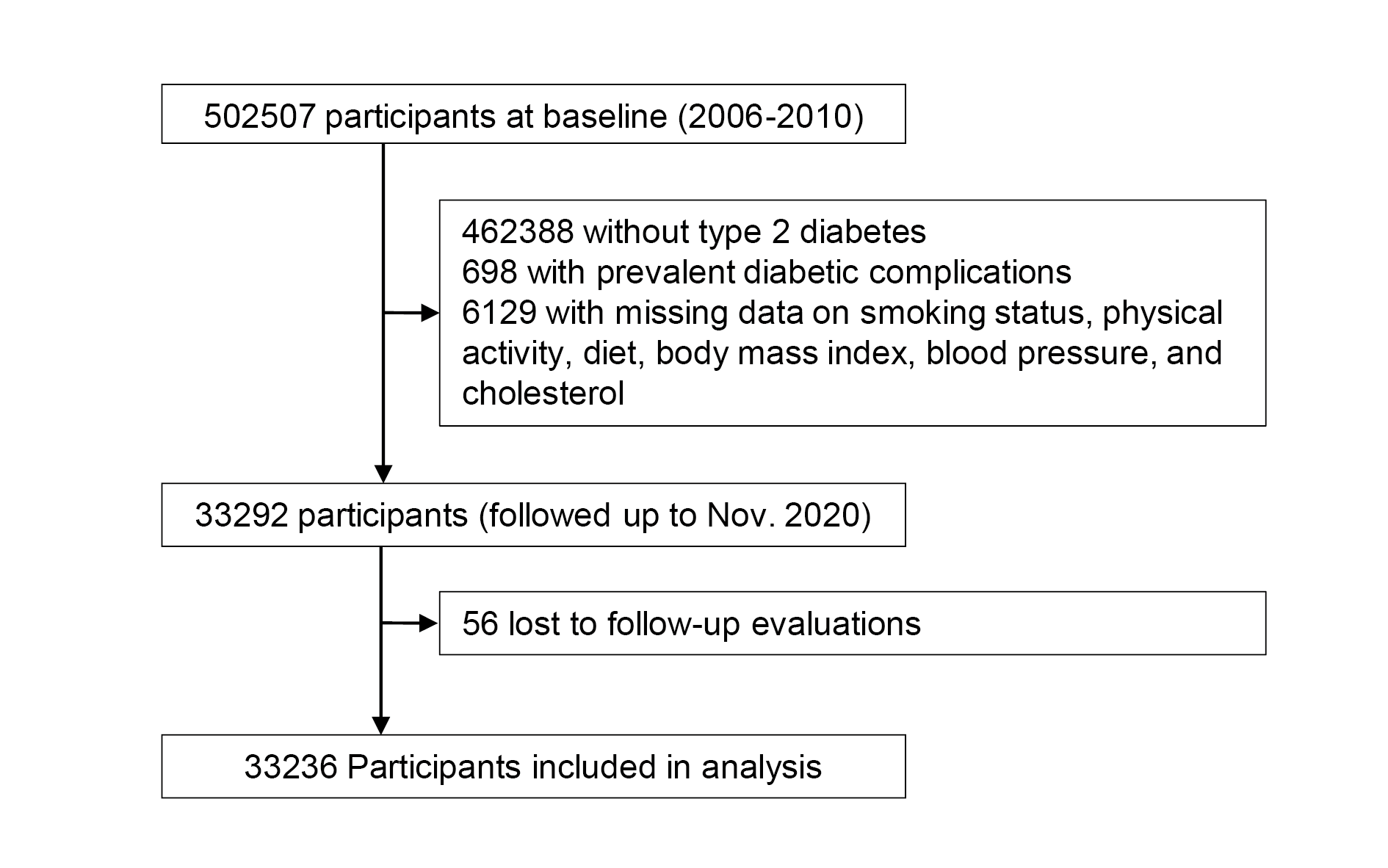


**Figure S1. Flowchart for the selection of the analyzed study sample from the UK Biobank study**


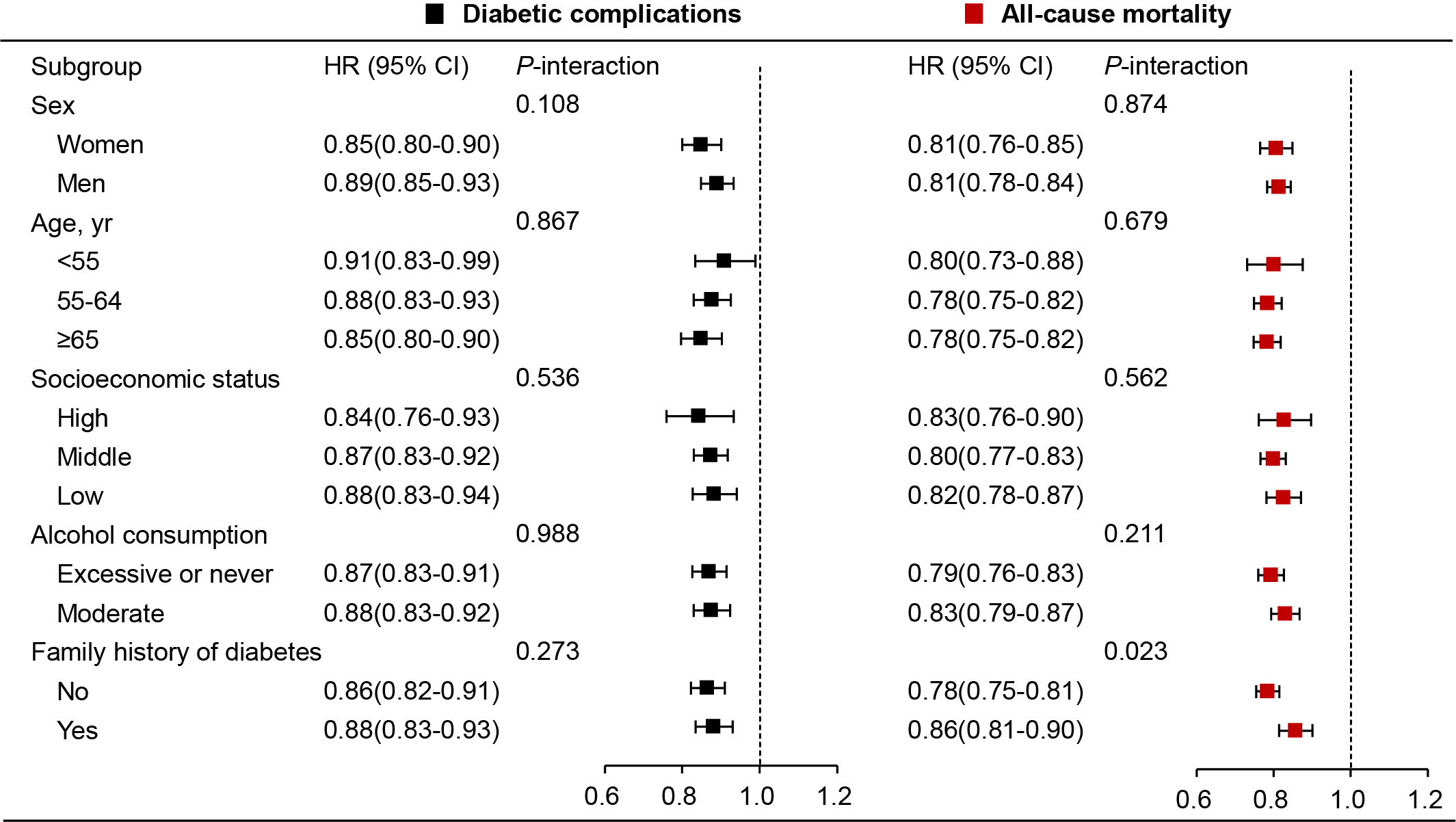


**Figure S2. Hazard ratios (HRs) of diabetic complications and all-cause mortality per 1-number increment in ideal cardiovascular health (CVH) metrics according to stratification categories among participants with type 2 diabetes.**

*Note*: Model was adjusted for sex, age, education level, socioeconomic status, ethnicity background, alcohol consumption, sugar-sweetened beverages, family history of diabetes, triglyceride, serum creatinine, and C-reactive protein.


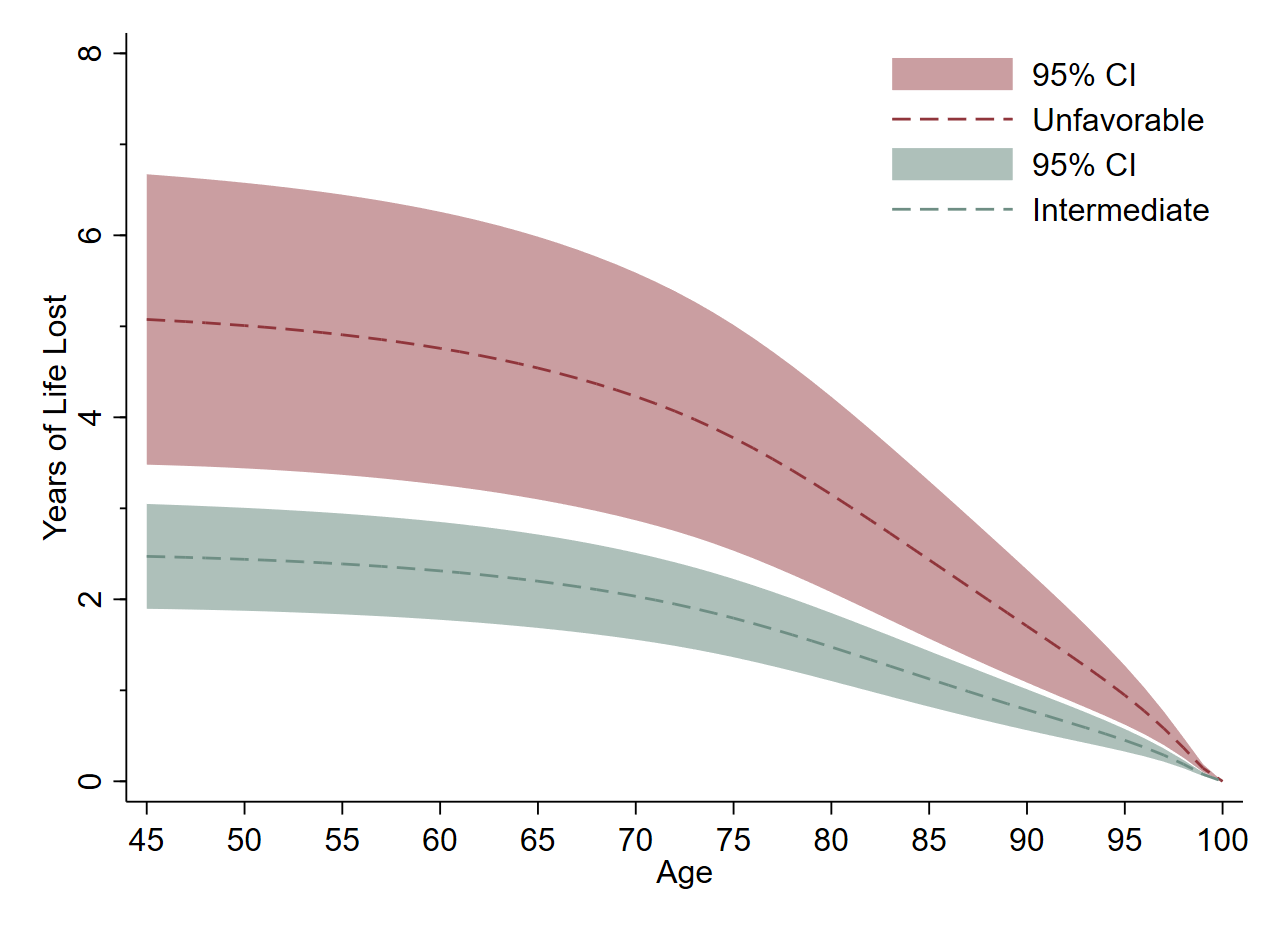


**Figure S3. Years of life expectancy lost by cardiovascular health (CVH) metrics among people with type 2 diabetes after further adjustment for diabetes duration.**

Note: Model was adjusted for sex, age, education level, socioeconomic status, ethnicity background, alcohol consumption, sugar-sweetened beverages, family history of diabetes, triglyceride, serum creatinine, C-reactive protein, and diabetes duration.


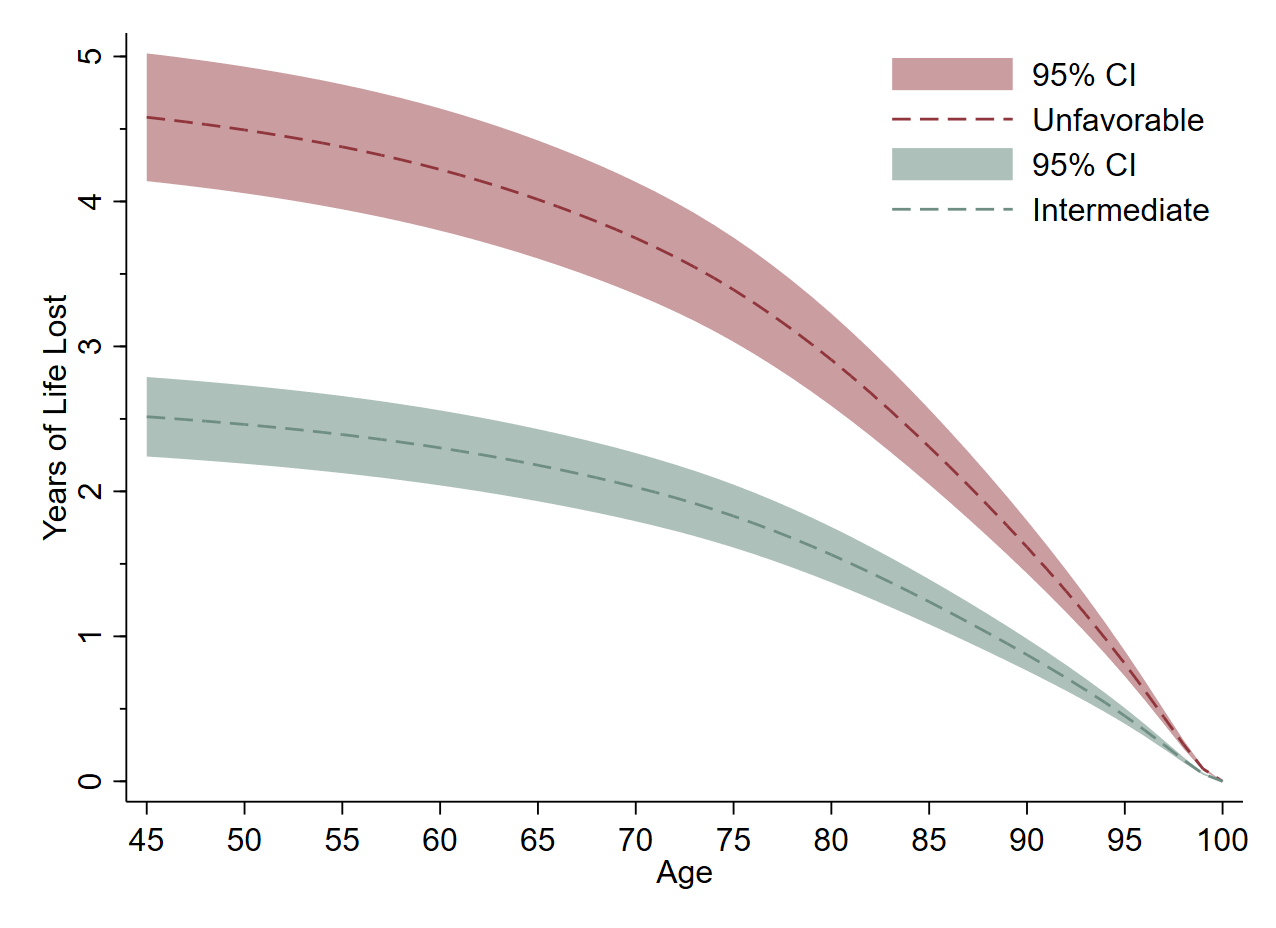


**Figure S4. Years of life expectancy lost by cardiovascular health (CVH) metrics among people with and without type 2 diabetes**

Note: Model was adjusted for sex, age, education level, socioeconomic status, ethnicity background, alcohol consumption, sugar-sweetened beverages, family history of diabetes, triglyceride, serum creatinine, C-reactive protein, and diabetes duration.
